# Ses: a Swin-Unet Edge-aware Segmentation network for uterine fibroid ultrasound images

**DOI:** 10.1186/s12938-026-01575-w

**Published:** 2026-05-04

**Authors:** Xiaotong Wang, Liling Shi, Wenjuan Wang, Lijuan Guo

**Affiliations:** 1 Children’s Hospital of Shanxi & Women Health Center of Shanxi, Taiyuan, 030013 Shanxi China; 2 Shanxi International Travel Healthcare Center (Taiyuan Customs Port Clinic), Taiyuan, 030021 Shanxi China

**Keywords:** Uterine fibroids, RCAN, RCF, Ultrasound image segmentation

## Abstract

Uterine fibroids represent one of the most prevalent gynecological tumors; however, their ultrasound images frequently exhibit indistinct boundaries and complex morphologies, thereby complicating accurate segmentation. An enhanced Swin-Unet-based framework, designated the Swin-Unet Edge-Sensitive Segmentation (SES) network, is proposed herein to advance boundary delineation and segmentation accuracy. The SES network incorporates the Residual Channel Attention Network (RCAN) to recalibrate feature responses via channel attention weighting, thereby reinforcing the representation of lesion regions, and the Richer Convolutional Features (RCF) module to preserve multi-scale spatial information through hierarchical feature integration, effectively addressing pixel-level classification in regions with blurred boundaries. The model was evaluated on annotated ultrasound images provided by Shanxi Provincial Children’s Hospital. Experimental findings demonstrate that SES consistently outperforms established architectures, including U-Net, U-Net++, Attention U-Net, and TransUNet, achieving superior performance across multiple indices (Dice coefficient: 0.9452; IoU: 0.8721; accuracy: 0.9358). Ablation analyses further substantiate the pivotal contributions of the RCAN and RCF modules to the overall segmentation performance. The proposed SES framework integrates global modeling capacity, multi-scale attention mechanisms, and edge-sensitive feature extraction to deliver a more accurate and robust solution for the ultrasound image segmentation of uterine fibroids, highlighting its substantial potential for clinical application.

## Introduction

Uterine fibroids are among the most common benign tumors of the female reproductive system, predominantly affecting women of childbearing age. Although generally non-malignant, they are frequently associated with symptoms, such as abnormal uterine bleeding, menstrual irregularities, pelvic or abdominal pain, and infertility. Originating from uterine smooth muscle cells, these benign gynecological tumors present considerable clinical challenges owing to their high prevalence and their profound impact on women’s reproductive health and overall quality of life [1, 2]. The precise etiology of uterine fibroids remains unclear; however, current evidence suggests a potential association with mutations in normal uterine myometrial cells as well as complex interactions between sex hormones and local growth factors [3]. In clinical practice, accurate diagnosis and precise localization of uterine fibroids are essential for selecting appropriate treatment strategies. Failure to detect and manage fibroids at an early stage may not only increase the risk of malignant transformation and adversely affect patients’ quality of life but also impose a considerable socioeconomic burden on patients and their families.

Ultrasonography is currently regarded as the most effective modality for the detection and localization of uterine fibroids. Nevertheless, in complex cases, its diagnostic performance still depends heavily on expert manual interpretation [4]. The development of automated techniques for the identification of uterine fibroids in ultrasound images is crucial for enhancing diagnostic efficiency and alleviating the workload of medical staff. At the same time, such approaches mitigate the influence of subjective factors on diagnostic outcomes, thereby rendering the process more objective and reliable. By strengthening the capacity for early detection, automated methods can facilitate timely treatment and intervention, prevent disease progression, and ultimately improve therapeutic outcomes and quality of life for patients.

With the rapid advancement of computer technology, computer-aided diagnosis has been increasingly applied to medical image analysis and has achieved notable success in areas, such as the recognition of uterine fibroids [5, 6]. Early approaches to uterine image segmentation primarily relied on traditional techniques, such as level set methods and C-means clustering. Carmelo [7] proposed a fully automatic segmentation method for the uterus and uterine fibroids based on unsupervised fuzzy C-means clustering and an iterative optimal threshold selection algorithm, which can automatically evaluate the boundaries and volumes of ablated fibroid regions without any external user input. Sarah et al. [8] developed a semi-automatic stacked-ellipse algorithm: the elliptical contour is manually initialized on a single sagittal ultrasound image, then fitted and deformed along the uterine half-axis to the transverse plane to achieve three-dimensional uterine segmentation. This method requires no training data and relies on geometric models and ultrasonic features. Ning et al. [9] designed a morphological active contour model independent of edge-based constraints, which is capable of acquiring accurate, real-time and non-rigid contours of ultrasonic lesions. Accelerated by GPU, the model runs at over 30 frames per second and thus supports real-time intraoperative tracking.

However, traditional approaches continue to encounter limitations, including imprecise delineation of fibroid boundaries and substantial inter-patient variability. In contrast, Convolutional Neural Networks (CNNs), with their superior feature extraction capabilities, have emerged as powerful tools, offering a range of effective solutions for uterine fibroid image segmentation. Zhang et al. [10] introduced a Mask-guided Hierarchical Learning (MHL) framework based on Fully Convolutional Networks (FCNs) for breast tumor segmentation. Dilma et al. [11] designed the MBF–CDNN network, which achieved an accuracy of 94.7% in classifying and detecting uterine fibroids from a dataset of 259 images. Kurata et al.[12] applied the U-Net architecture to uterine fibroid segmentation and reported improved performance through structural optimization and parameter tuning. Ning et al. [13] developed a three-stage neural network that integrates multi-dimensional information from MRI sequences (sequence scale), anatomical structures (anatomical scale), and pixel-level features while adopting a pyramid network structure to enhance segmentation accuracy. Zhang et al. [14] advanced this line of research by proposing an HIFUNet-based system for uterine fibroid MRI segmentation, employing ResNet101 [15] a for feature extraction and transposed convolution for upsampling. Liu et al. [16] proposed a 3D DA-VNet network integrated with deep supervision and attention mechanisms, which achieves accurate segmentation of uterine fibroids in MRI images by leveraging convolutional features.

With the advancement of Transformer architectures in computer vision, they have been increasingly applied to uterine fibroid segmentation to overcome the limitations of traditional CNNs in capturing long-range contextual dependencies. Some studies have enhanced U-Net by substituting CNN-based feature blocks with Transformer encoders, thereby leveraging self-attention mechanisms to model global semantic correlations and improve boundary delineation. Others have adopted hybrid architectures that integrate CNNs and Transformers, where CNNs extract local fine-grained features, while Transformers capture global structural information. This complementary design not only optimizes overall segmentation performance but also demonstrates superior robustness in cases involving fibroids with blurred boundaries or irregular morphologies, thereby achieving more accurate and reliable image segmentation. Zhao et al. [17] developed a dual-branch network that employs convolutional neural networks and Transformer networks separately to extract features from hysteroscopic images of uterine fibroids for classification, achieving an accuracy of 88.93%. Cao et al. [18] proposed a Swin Transformer-based segmentation model with a U-shaped structure and shifted window attention, achieving strong performance in classification tasks.

Current algorithms for uterine fibroid ultrasound image segmentation face several challenges. Traditional CNN-based methods (e.g., U-Net and its variants) are constrained by local convolutional operations, limiting their ability to capture long-range dependencies and reducing accuracy in delineating blurred boundaries. Many existing models also lack adaptive mechanisms for multi-scale feature fusion, which weakens their capacity to address the morphological variability of fibroids. Although Transformer-based approaches (e.g., TransUNet) can model global context, they remain less effective in capturing fine local details, while attention-driven networks (e.g., Attention U-Net) often struggle to balance global and local features. These limitations hinder the achievement of both segmentation accuracy and computational efficiency in clinical applications, particularly for ultrasound images with indistinct boundaries and diverse morphologies. Addressing these issues—boundary ambiguity, insufficient multi-scale feature fusion, and the balance between global and local representations—remains an important direction for advancing medical image segmentation.

The Swin-Unet Edge-aware Segmentation (SES) network is proposed in this study as an advanced model for uterine fibroid ultrasound image segmentation, specifically designed to address critical challenges, such as boundary ambiguity, inadequate multi-scale feature fusion, and the difficulty of balancing segmentation accuracy with computational efficiency. The framework integrates a Residual Channel Attention Network (RCAN) module to enhance feature representation and incorporates an edge-aware component, Rich Convolutional Features (RCF), to preserve fine-grained structural information. By combining these modules within the Swin-Unet architecture, the SES network achieves substantial improvements in delineating the complex characteristics of uterine fibroid ultrasound images while maintaining computational efficiency.

## SES model

**Fig. 1:**
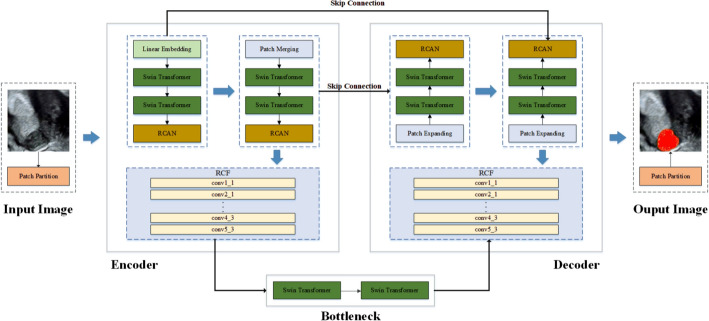
Framework of the proposed SES Model

The SES model is constructed on a Swin Transformer-based backbone and achieves accurate segmentation of uterine fibroid ultrasound images through hierarchical feature extraction and advanced fusion strategies, as illustrated in Fig. 1. The encoder employs Window-based Multi-Head Self-Attention (W-MSA) and Shifted Window Multi-Head Self-Attention (SW-MSA) [19] to capture both local and global dependencies. A Residual Channel Attention Network (RCAN) is embedded to refine spatial and channel representations, enhancing lesion-related features.

To address blurred boundaries, an edge-aware Rich Convolutional Features (RCF) branch [20] is added after the second-stage encoder. This module constructs a multi-scale edge feature pyramid using the five convolutional stages of VGG16 [21]. Each layer is compressed, upsampled, and independently supervised, ensuring effective learning of edge details. The bottleneck employs Swin Transformer layers for further refinement, and the decoder, symmetric to the encoder, restores resolution via upsampling and skip connections. During feature fusion, encoder–decoder features are combined with edge priors from RCF, substantially improving boundary delineation. The final segmentation map is generated through linear projection.

SES incorporates several core innovations, including the Swin Transformer backbone, the RCF edge-aware module, and the RCAN module. The main technical aspects of these components are described as follows:

- The Swin Transformer adopts a window-based self-attention mechanism, which processes local features via Window-based Multi-Head Self-Attention (W-MSA) and establishes cross-window connections through Shifted Window-based Multi-Head Self-Attention (SW-MSA). While ensuring computational efficiency, it effectively captures long-range dependencies, endows the model with robust feature extraction capability, and lays a solid foundation for constructing efficient multi-scale global context modeling.
- The Edge-Aware Residual Channel Feature (RCF) module constructs a multi-scale feature pyramid, strengthens edge feature learning through hierarchical supervision, and deeply fuses with the features of the backbone network, thereby optimizing the segmentation effect of blurred boundaries in ultrasound images in a targeted manner.
- The Residual Channel Attention Network (RCAN) module operates synergistically through dual attention branches (spatial and channel). The spatial branch focuses on multi-scale feature extraction, and the channel branch dynamically adjusts feature weights, which enhances the model’s ability to distinguish and capture complex lesion morphologies and micro-lesions.

### Dataset

The dataset used in this study was collected from Shanxi Provincial Children’s Hospital and consists of 372 original ultrasound images and 372 corresponding annotated images. Ethical approval was obtained from the institutional review board (ID: IRB-WZ-2024-001). To ensure dataset validity, both normal and abnormal cases were included, with all abnormalities confirmed by pathological examination. The annotated images were manually labeled and subsequently verified by experienced radiologists to ensure consistency and accuracy of the annotation positions. Meanwhile, this study adopts image blurring, flipping, rotation and noise injection to augment the image dataset. Specifically, global blurring is applied to the images in the blurring operation; horizontal flipping and vertical flipping are performed for image flipping; the images are rotated by $30^{\circ }$, $60^{\circ }$, $210^{\circ }$ and $240^{\circ }$ respectively, in the rotation process; and Gaussian noise with two intensity levels (mild and severe) is injected into the images during noise injection. The final dataset contains 3720 sample images.

**Fig. 2:**
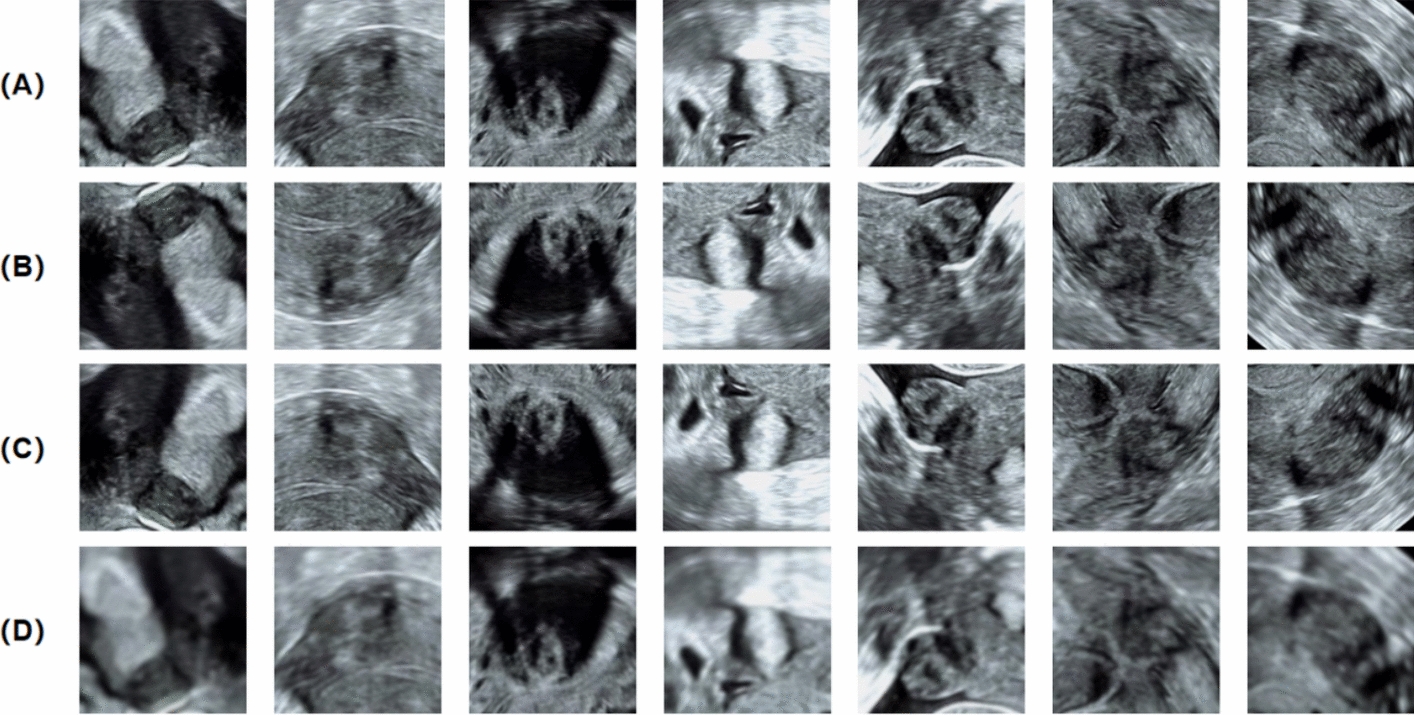
Image preprocessing

After augmentation, the final dataset comprised 3,720 images. Representative preprocessed samples are shown in Fig. 2, where row (A) displays the original images, rows (B) and (C) present flipped versions, and row (D) shows blurred images derived from the original dataset.

### Swin-Unet

**Fig. 3:**
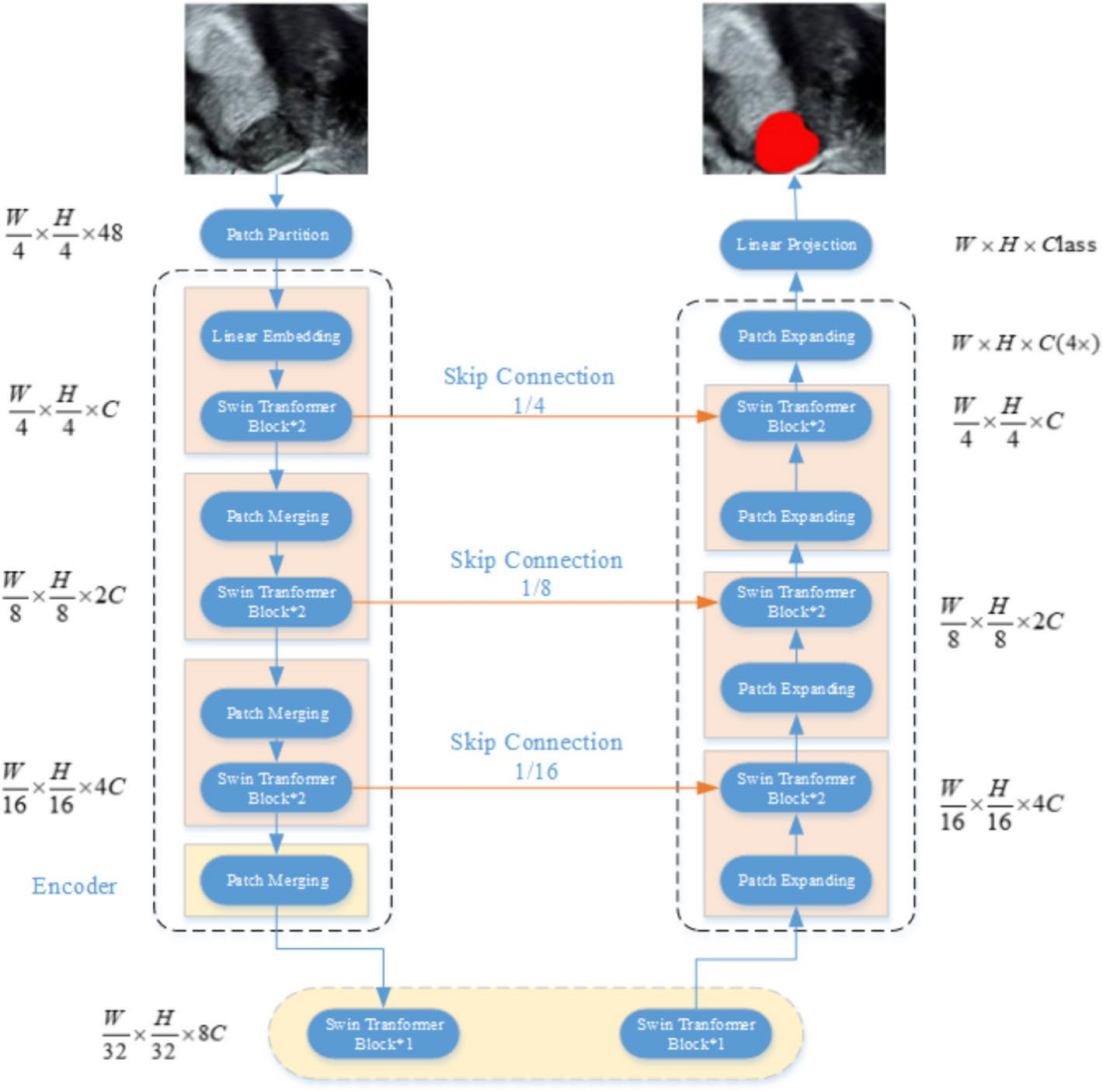
Overall architecture of the Swin-Unet model

Swin-Unet, illustrated in Fig. 3, is a Transformer-based segmentation network that combines the strong representational capacity of the Swin Transformer with the encoder–decoder design of U-Net, making it well-suited for fine-grained medical image segmentation tasks [22]. The architecture consists of four main components: encoder, bottleneck, decoder, and skip connections.

In the encoder, input images are processed through multiple Swin Transformer blocks that progressively extract features and reduce spatial resolution, generating high-dimensional representations. The bottleneck contains two Transformer layers that further integrate global features and enhance contextual modeling while maintaining feature dimensionality and resolution. The decoder, symmetric to the encoder, restores spatial resolution through stepwise upsampling. Skip connections fuse high-resolution shallow features with semantically rich deep features, enabling multi-scale feature integration and improving detail preservation [23, 24]. In the decoder part, the model adopts a Swin Transformer structure symmetric to the encoder, and gradually restores the spatial resolution of the feature map through progressive upsampling operations to achieve accurate image segmentation or reconstruction. To further improve the detail recovery capability, the model introduces a skip-connection mechanism, which fuses the high-resolution features from the shallow layers in the encoding path with the semantically rich features from the deep layers in the decoding path in a multi-scale manner. This effectively compensates for the spatial detail information lost during downsampling, thereby enhancing the edge-preserving ability and the accuracy of local structure reconstruction.

The SES model takes Swin-Unet as its core backbone network, fully leveraging the advantages of Swin Transformer in global modeling and hierarchical feature learning, thus providing a strong feature representation foundation for the subsequent construction of RCF and RCAN.

The Swin Transformer serves as the core module, introducing a window-based self-attention mechanism composed of Window Multi-Head Self-Attention (W-MSA) and Shifted Window Multi-Head Self-Attention (SW-MSA). W-MSA computes local attention within non-overlapping windows, improving efficiency through parallel computation. SW-MSA extends this by shifting windows, enabling cross-window dependencies and enhancing global contextual modeling. Each block first applies W-MSA for local features, followed by SW-MSA to expand contextual representation.

**Fig. 4:**
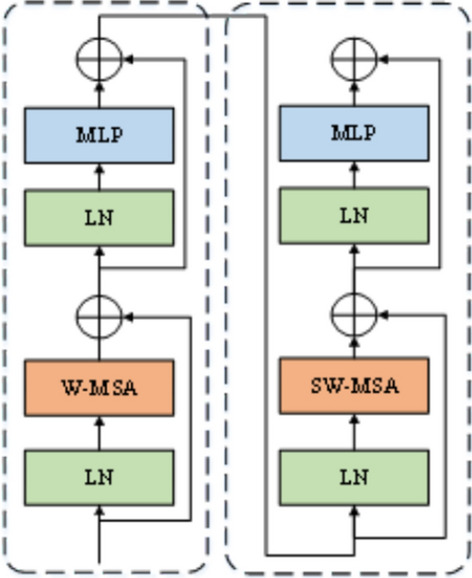
Swin transformer module diagram

Through this hierarchical design, Swin-Unet effectively captures both fine-grained local details and long-range dependencies, which is advantageous for medical image segmentation. A schematic of two cascaded Swin Transformer modules is shown in Fig. 4. The formulas for W-MSA and SW-MSA are shown in Eqs. (1)–(4).

1 $$\begin{aligned} \overline{z}^{l}=W-MSA(LN(z^{l-1}))+z^{l-1} \end{aligned}$$

2 $$\begin{aligned} z^{l}=MLP(LN(\overline{z}^{l}))+\overline{z}^{l} \end{aligned}$$

3 $$\begin{aligned} \overline{z}^{l+1}=SW-MSA(LN(z^{l}))+z^{l} \end{aligned}$$

4 $$\begin{aligned} z^{l+1}=MLP(LN(\overline{z}^{l+1}))+\overline{z}^{l+1} \end{aligned}$$

where LN denotes the layer normalization operation, and MLP refers to a multi-layer perceptron with a GELU activation function. Symbols $\overline{z}^{l}$ and ${z}^{l}$ respectively, represent the outputs of the SW-MSA and the MLP modules, respectively, in the *l*th block.

With Swin-Unet as its backbone, the SES model fully exploits the advantages of the Swin Transformer in global modeling and hierarchical feature learning. This provides a robust foundation for the subsequent integration of the RCF and RCAN modules, thereby reinforcing both edge perception and feature refinement capabilities.

### Rich convolutional features

**Fig. 5:**
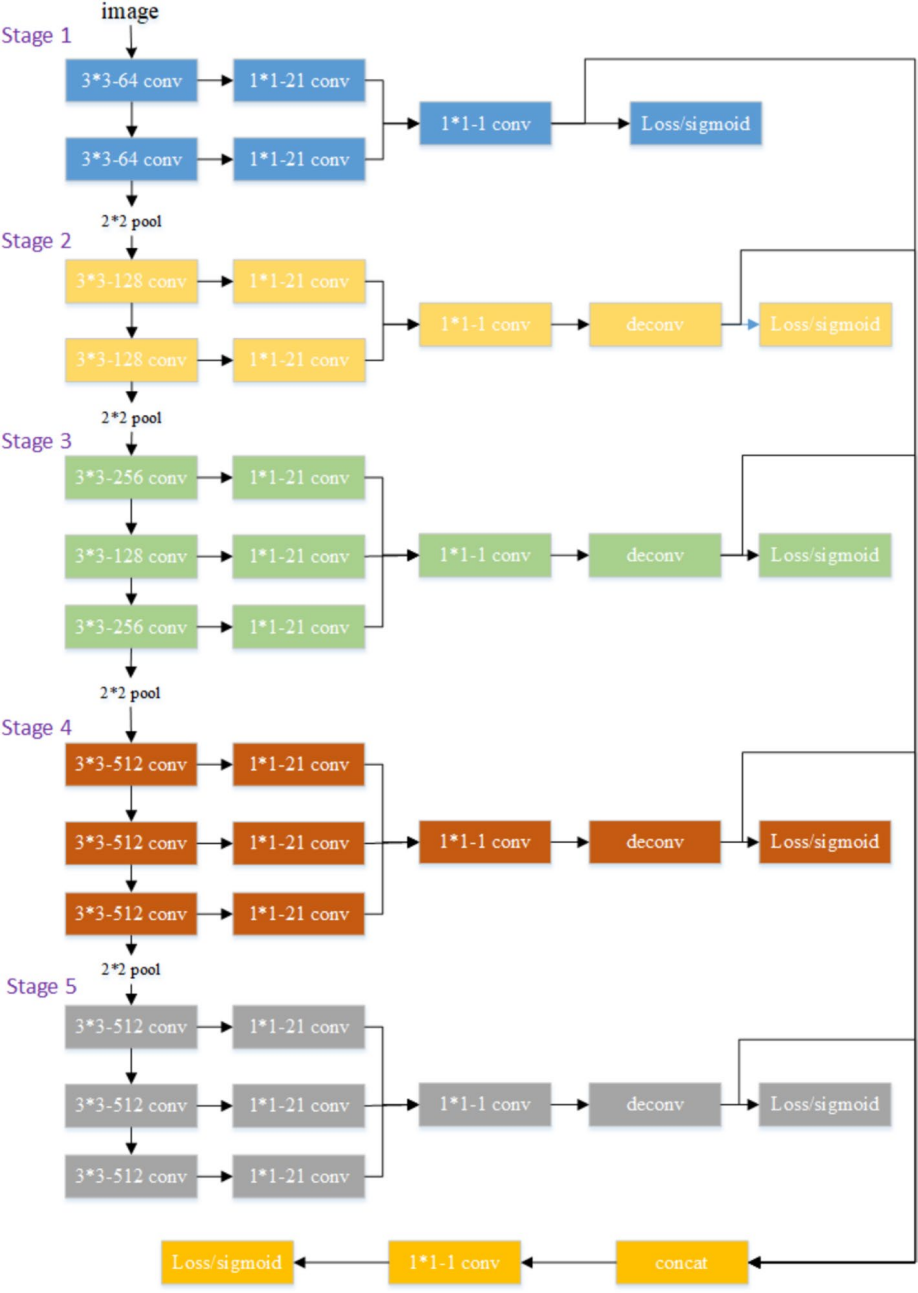
Architecture of the RCF model

The Rich Convolutional Features (RCF) model is an edge detection framework built on a fully convolutional network. It enhances edge detection accuracy by integrating multi-scale feature fusion with pixel-level edge awareness, and is particularly effective in capturing fine-grained edges and complex texture structures. This capability allows the model to focus more precisely on the edge regions of uterine fibroids. The edge-aware performance of RCF primarily depends on three components: multi-scale feature fusion, pixel-level edge supervision, and non-maximum suppression in the post-processing stage. The overall structure is illustrated in Fig. 5.

In RCF, the low-level features (Conv1–Conv3) capture fine-grained information, such as edges and textures, whereas the high-level features (Conv4–Conv5) capture semantic-level contours of objects. The feature extraction process is based on the VGG-16 backbone combined with ReLU activation functions. For the *j*th convolutional layer in the *i*th convolutional block—for example, Conv1, where *i* = 1 contains two convolutional layers—the corresponding mathematical formulations are expressed as follows:

5 $$\begin{aligned} C_{i,j}=\textrm{ReLU}(\textrm{Conv}(C_{i,j-1},k=3,c=C_{i,j})+b_{i,j}) \end{aligned}$$

where $C_{i,j}$ denotes the input of the *i*th convolutional block; $C_{i,j-1}$ represents the input of ${j-1}$th layer within *i*th block; $c=C_{i,j}$ indicates the number of output channels of the convolutional layer, and $b_{i,j}$ denotes the corresponding bias term.

Each convolutional block produces a feature map $C_{i}$ that aggregates information from all layers within the block. Shallow blocks (e.g., Conv1–Conv3) preserve high resolution and capture fine-grained details, such as edges and textures, while deeper blocks (e.g., Conv4–Conv5) generate lower-resolution maps with stronger semantic features, such as object contours. These complementary representations form a solid basis for multi-scale feature fusion.

Pixel-level edge supervision employs a multi-side output joint supervision strategy, which enhances the model’s ability to discriminate between edge and non-edge pixels while mitigating the problem of class imbalance. The formulation is defined in the following equation:

6 $$\begin{aligned} L_{\textrm{total}}=L_{\textrm{fusion}}+\sum _{i=1}^{s}L_{i} \end{aligned}$$

where $L_{total}$ denotes the total loss of pixel-level edge supervision; $L_{fusion}$ represents the loss of the final fused feature map; and Li denotes the pixel-level loss of the *i*th side output. The total loss is obtained by summing the fused loss with all side-output losses. This design ensures that features at each convolutional stage focus on edge learning. It also allows continuous correction of pixel-level prediction errors, thereby improving edge detection accuracy.

Non-maximum suppression (NMS) post-processing plays a crucial role in refining edge detection results, and its formulation is defined as in the following equation:

7 $$\begin{aligned} O(x,y)= {\left\{ \begin{array}{ll} P(x,y) \\ 0 & \end{array}\right. } \end{aligned}$$

where *P*(*x*, *y*) denotes the final edge probability map obtained by applying the Sigmoid activation function to the fused features, and *O*(*x*, *y*) denotes the final edge map obtained after non-maximum suppression (NMS) post-processing.

The RCF module integrates multi-scale feature fusion, pixel-level edge supervision, and non-maximum suppression (NMS) to enhance edge representation. Shallow layers capture fine-grained details, such as textures and local edges, while deeper layers provide semantic cues related to contours and large-scale structures. Pixel-level edge supervision introduces side-output branches at each feature layer, where 1$\times$1 convolutions followed by a Sigmoid activation generate edge probability maps. Each branch is supervised independently using binary cross-entropy loss, which forces the network to learn edge-specific features at multiple depths and alleviates the imbalance between edge and non-edge pixels. The application of NMS further refines the predicted edge maps by suppressing redundant responses and preserving sharp boundary structures. Through this combined design, RCF improves the delineation of blurred or irregular boundaries and enhances the robustness of segmentation results in medical images.

### Residual channel attention network module

Compared with previous CNN-based methods, the Residual Channel Attention Network (RCAN) achieves greater network depth and delivers superior performance in super-resolution (SR) tasks. RCAN adopts a Residual-in-Residual (RIR) structure, which facilitates the construction of an extremely deep yet highly trainable network.

The architecture of RCAN consists of four main components: shallow feature extraction, deep feature extraction, an upscaling module, and a reconstruction module. Shallow feature extraction relies solely on convolutional layers to capture low-level features from the input. Deep feature extraction is realized through the RIR structure, in which multiple residual blocks are stacked to enable the network to learn fine-grained information and thereby improve image reconstruction quality. Within RIR, Long Skip Connections (LSC) and Short Skip Connections (SSC) help bypass redundant low-frequency information, allowing the main network to focus on more discriminative features [25]. This design not only increases the effective depth of RCAN but also enhances its ability to capture fine details, significantly improving performance in ultrasound image segmentation tasks.

**Fig. 6:**
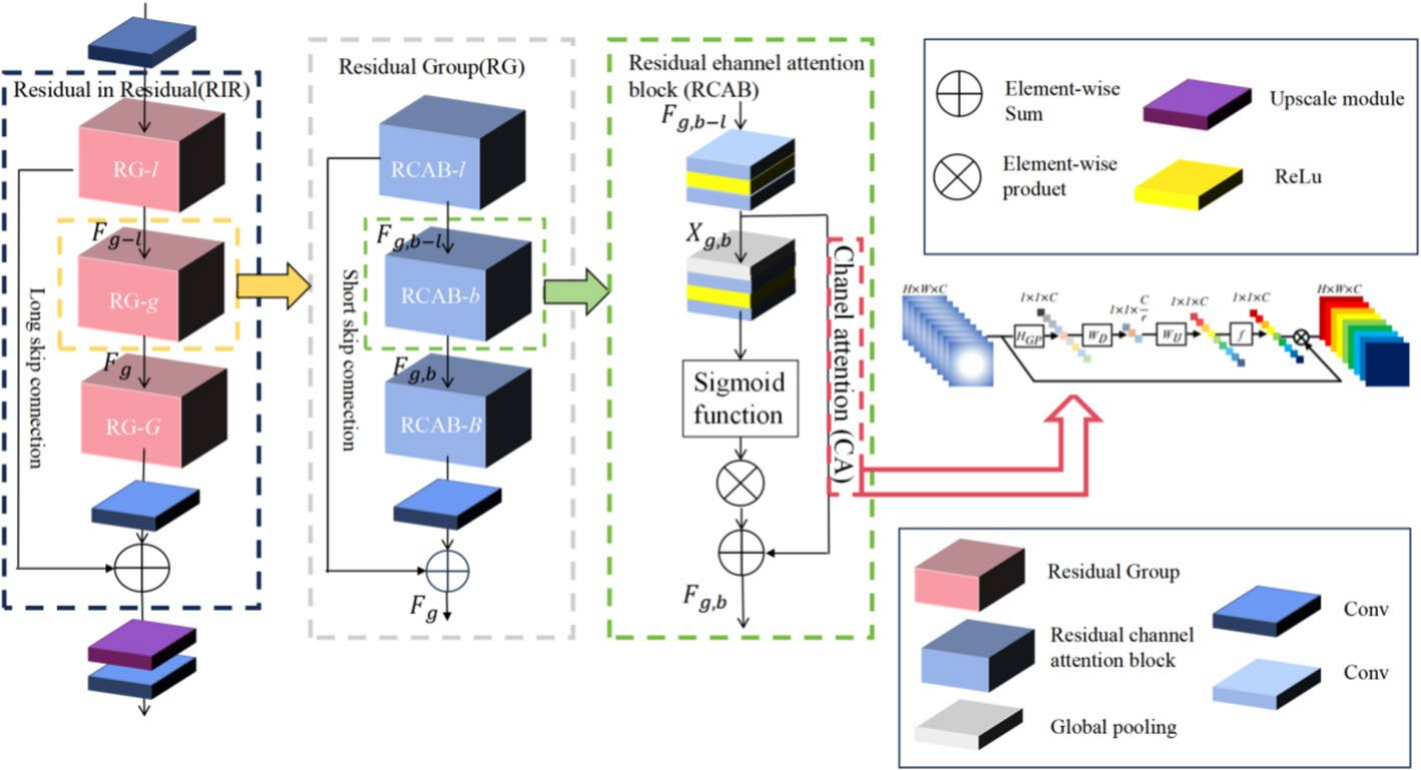
Architecture of the RCAN model

The upscaling module comprises multiple Residual Blocks (RBs), each incorporating a Channel Attention (CA) mechanism. The CA mechanism adaptively adjusts channel weights, enabling RCAN to automatically emphasize features most relevant to SR. Finally, the reconstruction module employs an upsampling strategy to transform feature representations into high-resolution outputs. The overall structure of RCAN is illustrated in Fig. 6.

Let $I_{LR}$ and $I_{SR}$ denote the input and output of RCAN, respectively. The shallow feature representation F0 is extracted from the input by applying a single convolutional layer, as expressed in the following equation:

8 $$\begin{aligned} F_{0}=H_{SF}(I_{LR}) \end{aligned}$$

where $H_{SF}(\cdot )$ denotes the convolution operation, and $F_0$ serves as the input for deep feature extraction in the subsequent RIR module. The formulation is further detailed in the following equation:

9 $$\begin{aligned} F_{DF}=H_{RIR}(F_{0}) \end{aligned}$$

where $H_{RIR}(\cdot )$ denotes the deep Residual-in-Residual (RIR) structure, which consists of *G* residual groups (RGs). The output of this structure is regarded as the deep feature representation.

The RIR structure is composed of G residual groups (RGs) together with long skip connections (LSCs). Each RG further contains B residual channel attention blocks (RCABs) with short skip connections (SSCs). This hierarchical design enables the training of extremely deep CNNs and facilitates high-resolution image super-resolution (SR). As the fundamental unit of the deeper network, the computation of an RG in the gth group is expressed in the following equation:

10 $$\begin{aligned} F_{g}=H_{g}(F_{g-1})=H_{g}(H_{g-1}(\cdots H_{1}(F_{0})\cdots )) \end{aligned}$$

where $H_g$ denotes the function of the *g*th residual group (RG), and $F_{g-1}$ and $F_g$ represent the input and output of the *g*th RG, respectively.

However, simply stacking multiple RGs does not guarantee improved performance. To address this limitation, the RIR structure introduces long skip connections (LSCs), which stabilize the training of very deep networks. Moreover, LSCs employ residual learning, as formulated in Eq. (11), to further enhance performance.

11 $$\begin{aligned} F_{DF}=F_{0}+W_{LSC}F_{G}=F_{0}+W_{LSC}H_{g}(H_{g-1}(\cdots H_{1}(F_{0})\cdots )) \end{aligned}$$

where $W_{LSC}$ denotes the weight of the convolutional layer at the end of the RIR structure, and the bias term is omitted here for simplicity. The introduction of LSC not only simplifies the flow of information between residual groups (RGs) but also enables the RIR to learn residual information at a coarse level.

The input features contain a substantial amount of information, while the objective of the super-resolution (SR) network is to recover the most useful components. A significant portion of low-frequency information can be bypassed using identity-based skip connections. Furthermore, each residual group (RG) contains B stacked residual channel attention blocks (RCABs). The computation of the *b*th RCAB in the *g*th RG is defined in the following equation:

12 $$\begin{aligned} F_{g,b}=H_{g,b}(F_{g,b-1})=H_{g,b}(H_{g,b-1}(\cdots H_{g,1}(F_{g-1})\cdots )) \end{aligned}$$

where $F_{g,b-1}$ and $F_{g,b}$ denote the input and output of the *b*th RCAB in the *g*th residual group (RG), respectively, and $H_{g,b}$ represents the corresponding transformation function.

To guide the main network toward focusing on more informative features, short skip connections (SSC) are introduced. The block output is thus obtained according to the following formulation, as expressed in the following equation:

13 $$\begin{aligned} F_{g}=F_{g-1}+W_{g}F_{g,B}=F_{g-1}+W_{g}H_{g}(H_{g,B}(\cdots H_{g,1}(F_{g-1})\cdots )) \end{aligned}$$

where $W_g$ denotes the weight of the convolutional layer at the end of the *g*th RIR. The introduction of SSC further enables the main network to learn residual information more effectively. By combining LSC and SSC, the training process can more easily filter out redundant low-frequency information, thereby improving the efficiency of feature learning.

## Experiment results and analysis

### Evaluation

The evaluation of segmentation performance was based on several metrics, including the Dice Similarity Coefficient, Intersection over Union (IoU), pixel-level accuracy, recall, and Hausdorff distance.

The Dice coefficient is employed to quantify the similarity between two sets. In image segmentation tasks, it evaluates the degree of overlap between the predicted region and the ground-truth annotation. The Dice coefficient is formally defined in the following equation:

14 $$\begin{aligned} \mathrm {Dice=}\frac{2\times \mid A\cap B\mid }{\mid A\mid +\mid B\mid } \end{aligned}$$

where $\mid A\mid$ denotes the number of pixels in the predicted segmentation region, $\mid B\mid$ denotes the number of pixels in the ground-truth region, and $\mid A\cap B\mid$ represents the intersection between the predicted segmentation and the ground truth, i.e., the number of correctly predicted pixels.

The Intersection over Union (IoU) is a widely used metric for evaluating segmentation accuracy. It is defined as the ratio between the intersection and the union of the predicted region and the ground truth, as expressed in the following equation:

15 $$\begin{aligned} \mathrm {IoU=}\frac{\mid A\cap B\mid }{\mid A\cup B\mid } \end{aligned}$$

where $\mid A\cup B\mid$ denotes the union of the predicted segmentation region and the ground truth, i.e., the total number of pixels in both regions. The IoU ranges from 0 to 1, with a value of 1 indicating complete overlap with the ground truth and 0 indicating no overlap.

Accuracy measures the ratio of correctly predicted pixels to the total number of pixels in the image, thereby reflecting pixel-level prediction performance. The formulation of accuracy is given in the following equation:

16 $$\begin{aligned} \mathrm {Accuracy=}\frac{\text {Correctly classified pixel count}}{\text {Total number of pixels}} \end{aligned}$$

Recall evaluates the model’s ability to correctly identify all true positive samples. In image segmentation, it represents the proportion of actual positive pixels that are correctly classified by the model. The formulation of recall is provided in the following equation:

17 $$\begin{aligned} \mathrm {Recall=\frac{TP}{TP+FN}} \end{aligned}$$

where TP (true positive) denotes the number of pixels correctly predicted as positive, and FN (false negative) denotes the number of pixels that are actually positive but incorrectly predicted as negative. A higher recall value indicates a stronger ability of the model to identify the target region.

The Hausdorff Distance (HD) measures the maximum distance between two sets, defined as the farthest distance from any point in one set to the nearest point in the other. In image segmentation, it is commonly used to assess boundary precision, particularly for evaluating edge details. The formulation of HD is given in the following equation:

18 $$\begin{aligned} H(A,B) = \max \left( \sup _{a \in A} \inf _{b \in B} \Vert a - b\Vert , \sup _{b \in B} \inf _{a \in A} \Vert a - b\Vert \right) \end{aligned}$$

### Comparative experiment

The proposed model was implemented in PyTorch (Python 3.9) and trained on an NVIDIA RTX 5060 GPU with 16 GB of memory. The initial hyper parameters for model training were set as follows: The proposed model was implemented in PyTorch (Python 3.9) and trained on an NVIDIA A100 GPU. The initial hyperparameters for model training were set as follows: batch size of 128, 100 training epochs, the Adam optimizer, and an initial learning rate of $1\times 10^{-3}$. To improve the generalization ability of the model, the Early Stopping algorithm was employed, with a patience value of 20. This approach terminates training automatically by monitoring performance on the validation set. Early Stopping provides two primary benefits: it substantially reduces computational resource consumption and effectively prevents model overfitting. Meanwhile, the SES model employs a composite loss function $L_{\text {total}} = L_{\text {Dice}} + L_{\text {BCE}} + \lambda \times L_{\text {RCF}}$, where $L_{\text {Dice}}$ is the Dice loss, $L_{\text {BCE}}$ is the binary cross-entropy loss, and $L_{\text {RCF}}$ is the sum of the side-output losses of the RCF edge branch. The weight $\lambda$ is set to 0.5.

For model evaluation, the dataset was partitioned into three subsets: a training set (75% of the data) for model learning, a validation set (20%) for monitoring generalization performance during training, and a test set (5%) for assessing final predictive performance. Furthermore, to improve the reliability of the model and the accuracy of the experimental results, K-fold cross-validation is employed for model training in this study. Raining was terminated when performance on the validation set ceased to improve, thus avoiding overfitting to the training data.

To evaluate the performance of SES, several segmentation methods including U-Net, U-Net++ [26], Attention U-Net, and TransUNet [27] are applied for comparative experiments, as shown in Table 1. The U-Net model adopts a classic four-layer encoder–decoder architecture with channel numbers of 64, 128, 256, and 512 for each layer, respectively, and employs transposed convolution for upsampling operations. The U-Net++ model retains the same depth as the U-Net model and is trained using deep supervision. The Attention U-Net model introduces an attention gate mechanism into the skip connections of U-Net to suppress irrelevant background regions and focus on the target regions. The TransUNet model adopts a hybrid CNN-Transformer architecture. Its encoder utilizes the CNN component of ResNet-50 to extract local features and captures global context via the Vision Transformer module, while its decoder is consistent with that of U-Net.

**Table 1:** Analysis of the results of the five models in the comparative experiment

Model	U-Net	U-Net++	Attention U-Net	TransUNet	SES
Dice	0.8903 ± 0.0125	0.9126 ± 0.0101	0.9265 ± 0.0087	0.9298 ± 0.0079	0.9452 ± 0.0061
IoU	0.7889 ± 0.0158	0.8256 ± 0.0124	0.8578 ± 0.0105	0.8544 ± 0.0096	0.8721 ± 0.0082
Accuracy	0.8955 ± 0.0102	0.9067 ± 0.0089	0.9253 ± 0.0073	0.9313 ± 0.0068	0.9358 ± 0.0054
Recall	0.8347 ± 0.0183	0.8576 ± 0.0183	0.8891 ± 0.0147	0.8893 ± 0.0121	0.9043 ± 0.0115
Hausdorff distance	3.3 ± 0.41	2.8 ± 0.35	2.5 ± 0.32	2.6 ± 0.29	2.0 ± 0.24

Table 1 shows that the SES model achieves the best performance, with a Dice coefficient of 0.9452, IoU of 0.8721, accuracy of 0.9358, recall of 0.9043, and Hausdorff distance of 2.0. TransUNet reaches a Dice coefficient of 0.9298 and IoU of 0.8544, while Attention U-Net attains 0.9265 and 0.8578, respectively. For the baseline models, U-Net and U-Net++ perform relatively lower, although U-Net++ exhibits a slight improvement over U-Net.

**Fig. 7:**
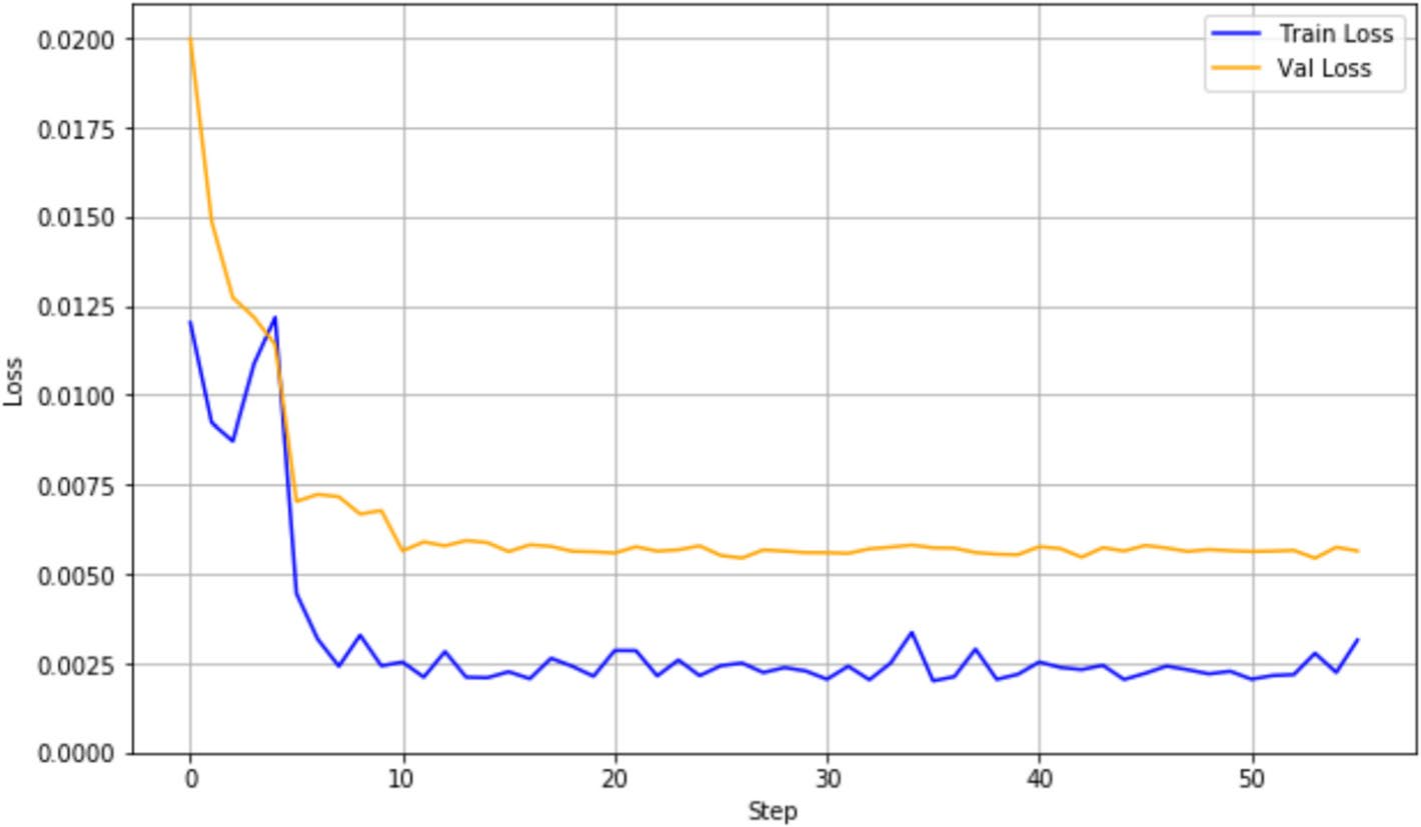
Training and validation loss curves of the SES Mode

To further assess the training process and convergence behavior of the proposed SES model, the variation of the loss function during training was recorded. Figure 7 presents the training and validation loss curves of the SES model. Both losses start at relatively high values and rapidly decrease within the initial training steps, after which they gradually stabilize. The training loss converges to a lower level, while the validation loss remains slightly higher but follows a similar trend. The close alignment between the two curves indicates effective convergence of the model without evident overfitting, demonstrating good generalization ability.

**Fig. 8:**
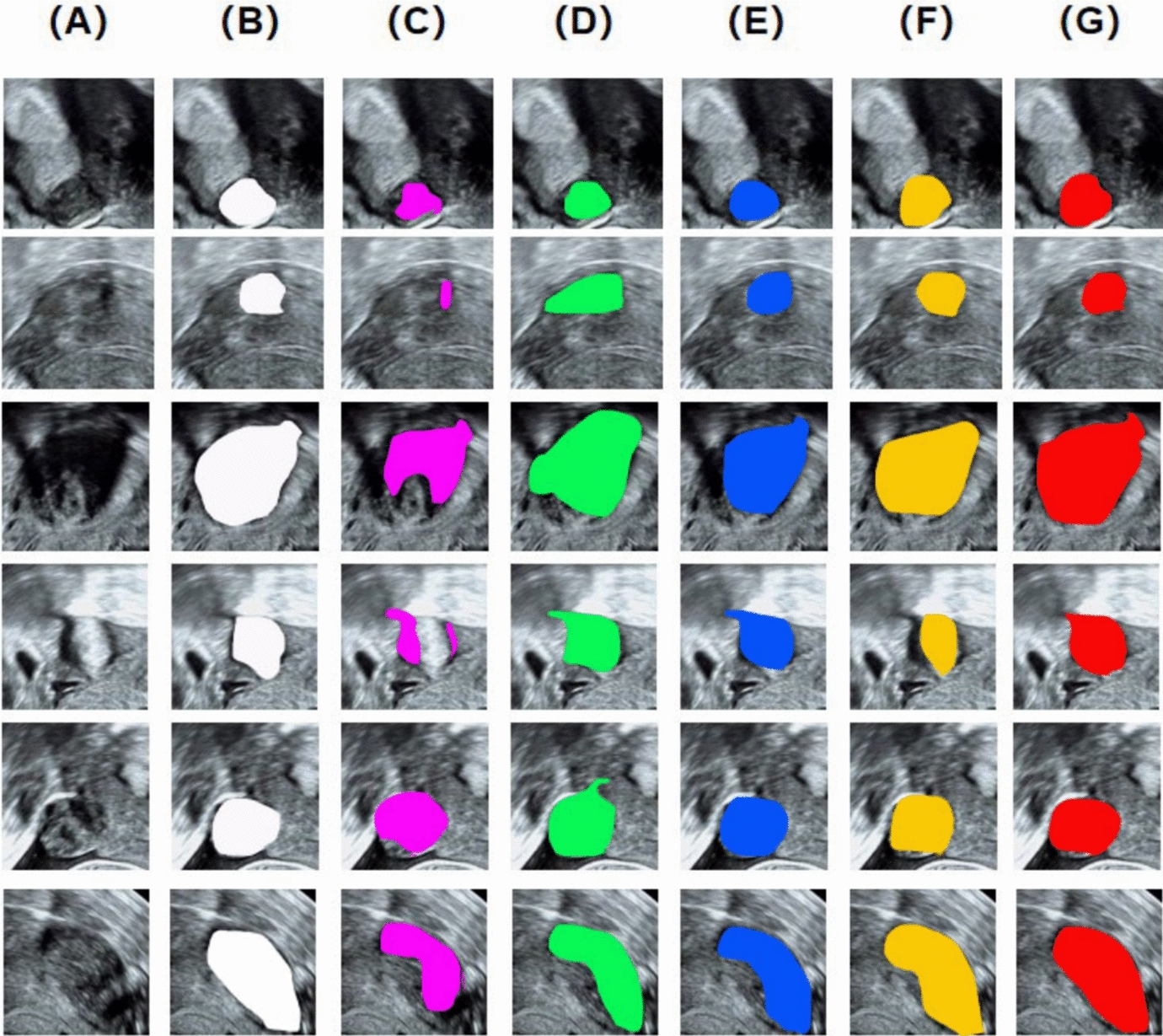
Segmentation results of uterine fibroid ultrasound images using different models: **A** Original image, **B** Ground truth, **C** U-Net, **D** U-Net++, **E** Attention U-Net, **F** TransUNet, and **G** SES

To more clearly demonstrate the segmentation performance of the SES model on uterine fibroid ultrasound images, Fig. 8 presents representative results from the self-collected dataset. Specifically: (a) original ultrasound image, (b) ground truth annotation, (c) U-Net prediction, (d) U-Net++ prediction, (e) Attention U-Net prediction, (f) TransUNet prediction, (g) Swin-Unet prediction, and (h) SES prediction.

As shown in Fig. 8, U-Net (c) exhibits obvious under-segmentation and over-segmentation, with poor stability. U-Net++ (d) improves upon U-Net but still suffers from blurred boundaries. Attention U-Net (e) enhances edge recognition accuracy, particularly visible in the third row. TransUNet (f) benefits from global modeling capability and achieves good overall contour segmentation, but local discontinuities remain. In contrast, the proposed SES model (h), which integrates RCAN and RCF, significantly improves edge delineation and detail preservation, yielding segmentation results that closely align with the ground truth annotations.

Together with the quantitative results in Table 1, these visualizations confirm that the SES model can accurately identify uterine fibroid nodules of varying shapes and sizes, achieving segmentation performance highly consistent with expert annotations.

To evaluate whether the difference in the Dice coefficient between the SES model and other mainstream models (U-Net, U-Net++, Attention U-Net, TransUNet) is statistically significant, paired t tests were performed on the results of fivefold cross-validation in this study. As shown in Table 2, the differences between SES and U-Net, U-Net++ are highly significant (all *p* < 0.01), and the differences between SES and Attention U-Net, TransUNet are significant (*p* < 0.05). These results indicate that the performance improvement of the SES model is not caused by random fluctuation, but represents a statistically significant advantage in segmentation performance.

**Table 2:** Paired t test results for performance comparisons between different models

Model	Dice	*p* value (vs SES)
U-Net	0.8903 ± 0.0125	*p* < 0.01
U-Net++	0.9126 ± 0.0101	*p* < 0.01
Attention U-Net	0.9265 ± 0.0087	*p* < 0.05
TransUNet	0.9298 ± 0.0079	*p* < 0.05
SES	0.9452 ± 0.0061	–

### Ablation experiment

Ablation experiments were conducted to evaluate the contribution of each module within SES. The results are summarized in Table 3, where bold values indicate the best performance achieved in each setting.

**Table 3:** Comparison of ablation experiment results

Swin-Unet	RCAN	RCF	Dice	IoU	Accuracy	Recall	Hausdorff distance
$\checkmark$			0.8549	0.8012	0.8703	0.8285	3.3
$\checkmark$	$\checkmark$		0.8732	0.8159	0.8793	0.8454	3.1
$\checkmark$		$\checkmark$	0.8947	0.8374	0.8836	0.8521	2.5
	$\checkmark$	$\checkmark$	0.7946	0.7528	0.8528	0.8067	3.8
$\checkmark$	$\checkmark$	$\checkmark$	0.9452	0.8721	0.9358	0.9043	2.0

When the RCAN module was removed, the Dice coefficient decreased to 0.8947, indicating that this module contributes to improved feature attention and edge recognition. Excluding the RCF module further reduced the Dice coefficient to 0.8732, confirming its role in edge-guided supervision for boundary localization. Replacing the Swin-Unet [18] backbone with a conventional CNN led to a substantial decline in performance, demonstrating the positive impact of long-range dependency modeling. The CNN component of the Swin-Unet model is constructed based on the standard ResNet-50 architecture. The input single-channel ultrasound images are adapted via channel replication, and the network output is adjusted to channel numbers of [96, 192, 384, 768] through 1$\times$1 convolution to match the input specifications of Swin-Unet. Using only the Swin-Unet backbone yielded a Dice coefficient of 0.8549, which performed better than CNN substitution but remained lower than the full SES model.

These results show that each module—RCAN, RCF, and Swin-Unet—provides measurable improvements, with the full integration of all three producing the highest segmentation accuracy.

Furthermore, we conducted ablation experiments using various attention mechanisms on this basis, primarily employing the Squeeze-and-Excitation (SE) and Convolutional Block Attention Module (CBAM) mechanisms. The SE module adaptively recalibrates the feature responses between channels by modeling the interdependencies among channels. In contrast, the CBAM module introduces a dual attention architecture, which sequentially leverages and calibrates channel attention and spatial attention, enabling the model to focus on informative feature channels. Table 4 illustrates the comparative results of different attention mechanisms. The proposed SES model in this paper achieves significantly superior performance compared to the models integrated with the SE module and CBAM module. This advantage can be attributed to the dual attention design of the RCAN and its powerful multi-scale spatial feature extraction capability, which endows the model with better feature learning ability.

**Table 4:** Comparison of different attention mechanisms

Attention mechanism	Dice	IoU	Accuracy	Recall	Hausdorff distance
SE	0.8912	0.8325	0.8917	0.8612	2.8
CBAM	0.9024	0.8447	0.9035	0.8723	2.6
SES	0.9452	0.8721	0.9358	0.9043	2.0

### Activation map visualization

**Fig. 9:**
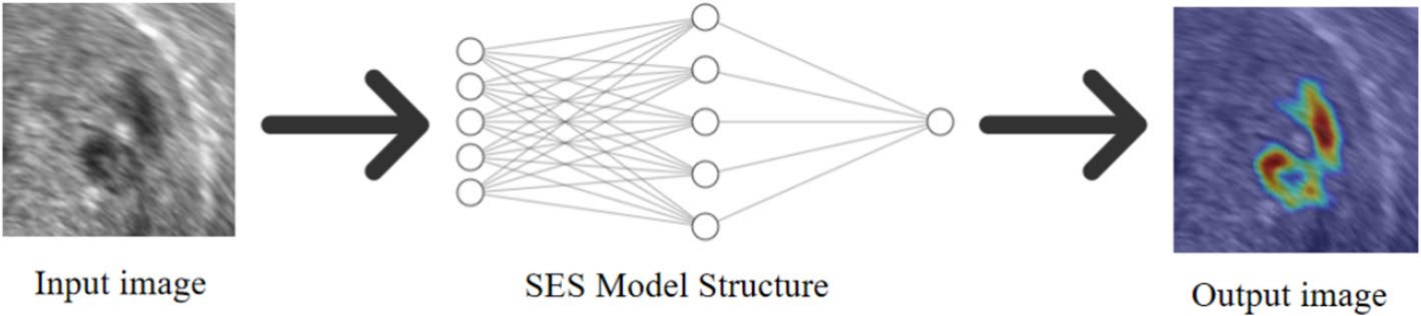
Module activation heatmap

To qualitatively validate the effectiveness of the SES model proposed in this study, we visualized the activation maps of the SES model using Grad-CAM. Due to the deeply embedded architecture of the RCAN and RCF modules, it is technically challenging to extract their activation maps individually. Therefore, we generated and analyzed the activation heatmaps of the complete SES model to indirectly demonstrate the synergistic effect of the two modules, as illustrated in Fig. 9. The results in Fig. 9 show that the activation heatmaps of the SES model exhibit intense and focused responses in the uterine fibroid region. Compared with the original ultrasound images, the heatmaps indicate that the model effectively suppresses irrelevant background noise and clearly delineates the boundaries of the tumor. The activation hotspots are highly consistent with the ground truth of the tumor, providing intuitive evidence that the SES model can capture clinically relevant features.

### Results analysis

We proposed the SES network for uterine fibroid ultrasound image segmentation and comprehensively evaluated its performance against several state-of-the-art methods. Both quantitative results and qualitative visualizations demonstrate that SES achieves superior segmentation accuracy, particularly in delineating blurred boundaries and preserving fine structural details. The ablation experiments further confirm the distinct contributions of the RCAN and RCF modules, as well as the effectiveness of the Swin-Unet backbone. These findings suggest that integrating multi-scale attention mechanisms with edge-aware feature learning provides a robust strategy for addressing the challenges of ultrasound image segmentation.

In the comparative experiments, Table 1 shows that the SES model achieved the highest overall performance across multiple metrics, including Dice coefficient, IoU, accuracy, recall, and Hausdorff distance. U-Net and U-Net++ performed relatively poorly, reflecting the limitations of their basic architectures. Although U-Net employs a symmetric encoder–decoder design with skip connections, its simple structure limits its ability to capture multi-scale information, leading to lower accuracy in complex boundary segmentation. U-Net++ improves feature fusion and gradient flow through nested skip connections and dense convolutional blocks, yielding slightly better results, but it does not fundamentally address deficiencies in multi-scale fusion and global context modeling. Attention U-Net and TransUNet outperformed the U-Net series but remained inferior to SES. Attention U-Net improves local feature extraction through the attention mechanism but lacks global context modeling, reducing its effectiveness in complex backgrounds or blurred boundaries. TransUNet leverages Transformer-based global modeling and performs well in contour segmentation, yet it shows weaknesses in local continuity, particularly for small targets and fine edge structures. Compared with these architectures, SES demonstrates clear advantages in segmentation accuracy and robustness. The RCAN module enhances feature representation through spatial and channel attention. The RCF module provides edge-aware supervision, improving boundary delineation and continuity of fine structures. The Swin-Unet backbone enables long-range dependency modeling and multi-scale feature learning, capturing both global context and local detail. Through the combined effect of these components, SES achieves strong performance in segmenting ultrasound images with ambiguous boundaries and variable morphologies.

In the ablation experiments, Table 3 shows that the complete SES model achieved the highest performance across all metrics, while the removal or replacement of individual modules resulted in varying degrees of decline. The significant drop in the Dice coefficient after removing the RCAN module indicates that RCAN plays a critical role in enhancing the model’s focus on edges and key regions. Without RCAN, the model had difficulty perceiving blurred boundaries, reducing segmentation accuracy. Further degradation after removing the RCF module confirms that edge-guided supervision is indispensable for precise boundary localization. When the Swin-Unet backbone was replaced with a conventional CNN, the performance declined sharply, underscoring the importance of long-range dependency modeling provided by the Swin Transformer. Using only the Swin-Unet backbone yielded better results than the CNN-based structure but remained inferior to the full SES model. This suggests that the Transformer backbone alone cannot fully optimize local details and edge segmentation, and that the synergistic contributions of RCAN and RCF are required to achieve optimal performance. Overall, the ablation results demonstrate that each module contributes distinct improvements, and their integration enables SES to maintain superior segmentation accuracy, particularly in handling blurred boundaries and fine structural details.

Meanwhile, we also analyzed the computational efficiency. The SES model adopted in this study achieves an excellent trade-off between segmentation performance and computational efficiency. Although its number of parameters is slightly larger than that of the U-Net model, it significantly outperforms all comparative methods in terms of segmentation accuracy. The inference time of the SES model is approximately 32.4 ms, which represents a median level for clinical diagnosis and can support real-time or near-real-time clinical diagnosis.

Although the SES model delivers superior overall performance, it still exhibits segmentation failures or reduced accuracy in specific challenging cases. When uterine fibroids appear isoechoic or show extremely weak echo differences relative to the surrounding normal myometrium on ultrasound images, the model struggles to distinguish boundaries using gray-scale information alone. This causes the boundary between the tumor and the uterine wall to become nearly indistinguishable, preventing the RCF module of the SES model from extracting edge features. Consequently, the SES model produces obvious under-segmentation in the predicted regions, with the Dice coefficient falling below 0.6. These observations demonstrate that relying solely on visual image features has certain limitations. In future research, introducing multimodal information of uterine fibroids may represent an effective approach to overcome these problems.

## Conclusion

Uterine fibroids often exhibit blurred boundaries and complex morphologies in ultrasound imaging, making accurate segmentation challenging. This study proposed the Swin-Unet Edge-aware Segmentation (SES) network, which integrates the Swin Transformer backbone with the Residual Channel Attention Network (RCAN) and the Rich Convolutional Features (RCF) branch. The design combines global contextual modeling, multi-scale feature fusion, and edge-aware supervision to improve segmentation accuracy and robustness.

Experimental results show that SES outperforms mainstream models, including U-Net, U-Net++, Attention U-Net, and TransUNet, with a Dice coefficient of 0.9452, IoU of 0.8721, accuracy of 0.9358, recall of 0.9043, and Hausdorff distance of 2.0. Ablation studies further confirm module contributions: removing RCAN reduced the Dice score to 0.8947, removing RCF decreased it to 0.8732, and replacing Swin-Unet with a CNN led to a sharp drop to 0.7946. These results indicate that each component contributes to performance, and their integration ensures optimal outcomes.

In conclusion, SES addresses limitations of existing CNN and Transformer-based architectures through attention-based refinement, edge-aware supervision, and hierarchical global modeling. It achieves precise and stable segmentation of uterine fibroid ultrasound images and shows potential as a reliable computer-aided diagnostic tool.

## Data Availability

No datasets were generated or analysed during the current study.
